# Segmental differences in Slc26a3-dependent Cl^−^ absorption and HCO_3_^−^ secretion in the mouse large intestine in vitro in Ussing chambers

**DOI:** 10.1186/s12576-020-00784-9

**Published:** 2021-01-29

**Authors:** Hisayoshi Hayashi, Hiroki Nagai, Kou-ichiro Ohba, Manoocher Soleimani, Yuichi Suzuki

**Affiliations:** 1 Laboratory of Physiology, School of Food and Nutritional Sciences, University of Shizuoka, 52-1 Yada, Suruga-ku, Shizuoka, 422-8526 Japan; 2 Department of Medicine, University of Cincinnati, Cincinnati, OH 45267 USA

**Keywords:** DRA, NaCl absorption, Congenital chloride-losing diarrhea, Feces

## Abstract

The anion exchanger slc26a3 (DRA), which is mutated in congenital chloride-losing diarrhea, is expressed in the apical membrane of the cecum and middle-distal colon but not in the proximal colon of rodent large intestines. To elucidate the functional roles of DRA, we measured unidirectional ^36^Cl^−^ and ^22^Na^+^ fluxes and HCO_3_^−^ secretion in vitro in each of these segments using DRA-KO mice. Robust Cl^−^ absorption, which was largely abolished after DRA deficiency, was present in the cecum and middle-distal colon but absent in the proximal colon. Na^+^ absorption was present in all three segments in both the control and DRA-KO mice. The luminal-Cl^−^-dependent HCO_3_^−^ secretions in the cecum and middle-distal colon were abolished in the DRA-KO mice. In conclusion, DRA mediates Cl^−^ absorption and HCO_3_^−^ secretion in the mouse cecum and middle-distal colon, and may have roles in H_2_O absorption and luminal acid/base regulation in these segments.

## Introduction

Intestinal fluid absorption is mediated by epithelial NaCl absorption involving parallel operation of Na^+^/H^+^ and Cl^−^/HCO_3_^−^ exchangers in the apical membrane and water followed by osmotic forces, which was first proposed based on in vivo perfusion experiments in humans [1]. This model indicated that congenital chloride-losing diarrhea (CCD), a disorder characterized by massive loss of chloride in acidic watery stools [2–4], is caused by a defect in the Cl^−^/HCO_3_^−^ exchanger [5, 6]. Genetic analyses have shown that mutations in human SLC26A3 (also known as DRA; downregulated in adenoma) result in CCD [7, 8]. The DRA-KO mouse model was reported to closely resemble human CCD in several important pathophysiological aspects [9].

Studies of both human and mouse DRAs in heterologous expression systems have demonstrated that they mediate Cl^−^/HCO_3_^−^ exchange, although some controversy remains concerning substrate specificity, stoichiometry and electrogenicity [10–13]. Functional studies in vivo or in isolated mucosa using DRA-KO mice or a mouse model of ileocolitis have shown that DRA is involved in Cl^−^ absorption and HCO_3_^−^ secretion, and plays an important role in Na^+^ and H_2_O absorption in the mouse large intestine [14–19].

The purpose of the present study is to further clarify the physiological roles of DRA in the mouse large intestine by separately examining each segment along the cecocolonic axis. Accumulating evidence has shown that the mucosal transport function of the mammalian large intestine is not uniform but dissimilar along the cecocolonic axis [20–25]. The expression of DRA and a Na^+^/H^+^ exchanger, NHE3, along the cecocolonic axis of the rodent intestine has been precisely examined: DRA expression was absent in the proximal colon and highest in the late middle colon, and it was also prominent in the cecum [24, 26]. In contrast, NHE3 expression was highest in the proximal colon and middle colon but negligible in the cecum and distal colon [24]. These expression profiles were consistent with the surface pH and water, Na, and Cl compositions of the luminal contents [24]. We therefore separated the large intestinal tissue into three segments: the cecum, proximal colon, and middle-distal colon. In each segment, we determined (1) unidirectional ^36^Cl^−^ and ^22^Na^+^ fluxes, (2) the Cl-dependent HCO_3_^−^ secretion rate, and (3) the following characteristics in the feces: H_2_O content, Na^+^, K^+^, and Cl^−^ concentrations, and pH. These experiments and measurements were also repeated using DRA-KO (slc26a3(−/−)) mice, and the results were compared with those obtained from non-DRA-KO control mice [slc26a3(+/+) and slc26a3(+/−)] to elucidate the functions of DRA.

## Materials and methods

### Animals

slc26a3(−/−) mice were originally generated in the laboratory of Prof Soleimani as described previously [9]. slc26a3+/+, slc26a3+/−, and slc26a3(−/−) (DRA-KO) mice bred on a C57BL/6J genetic background were obtained from breeding pairs housed at the University of Shizuoka. Mice were fed a standard pellet diet (MF, Oriental Yeast, Tokyo, Japan), and water was provided ad libitum. Genotype analysis of the offspring was performed by PCR of DNA isolated from tail snips. In the following experiments, both male and female mice ranging in age from 2 to 4 months were used. All animal experiments were approved by the Animal Care and Use Committee of the University of Shizuoka and conducted in accordance with the Guidelines and Regulations for the Care and Use of Experimental Animals by the University of Shizuoka.

### Measurement of ^36^Cl^−^ and ^22^Na^+^ fluxes and electrical parameters

The mice were killed by cervical dislocation, and the distal intestine from the ileum to the anus was removed. We used three segments: the cecum, the proximal colon that displays palm-leaf striations (0–30% length), and the middle-distal colon (30–100% length) (see the picture in Fig. 6). Each segment was isolated and opened, and luminal stool specimens were collected. The sample was then rinsed with buffer to remove the remaining intestinal contents, and the external muscle layer was removed by blunt dissection under a stereomicroscope. The tissue was then mounted vertically between Ussing chambers with an internal surface area of 0.2 cm^2^. The bathing solution in each chamber was 5 ml and was kept at 37 ℃ in a water jacketed reservoir. The bathing solution contained (in mM) 119 NaC1, 21 NaHCO_3_, 2.4 K_2_HPO_4_, 0.6 KH_2_PO_4_, 1.2 CaCl_2_, 1.2 MgCl_2_, and 10 glucose, and gassed with 95% O_2_ and 5% CO_2_ (pH 7.4). Tissues were continuously short-circuited using a voltage-clamping amplifier (CEZ9100, Nihon Kohden, Tokyo, Japan). The short-circuit current (Isc) value was expressed in μEq per square centimeter of tissue per hour and was positive when the positive charge flowed from the mucosa to serosa. Transmural tissue conductance (Gt) was calculated from the change in current in response to voltage pulses according to Ohm’s law and expressed as mS per square centimeter of tissue. The unidirectional fluxes of mucosal-to-serosal (Jms) and serosal-to-mucosal (Jsm) were measured in the adjacent tissues. After addition of a small amount of ^36^Cl and ^22^Na together (in some cases, either ^36^Cl or ^22^Na alone) to either the mucosal (M) or serosal (S) hemichamber, 30 min were allowed for the isotopic steady state to be reached. Samples (0.5 ml each) were then taken 3–6 times from the unlabeled side at 20-min intervals and replaced with an equal volume of the unlabeled solution. At the beginning and end of each experiment, 50 μl samples were taken from the “hot side” to obtain the mean specific activities of ^36^Cl and ^22^Na. Unidirectional fluxes were calculated from the changes in tracer activity on the unlabeled side after correcting for dilution by replacement after sampling and are expressed as μmol per square centimeter of tissue per hour. Isc, Gt and unidirectional fluxes thus obtained for each of 2–5 successive 20-min flux periods were averaged to establish the given results. The epithelial sodium channel (ENaC) blocker benzamil (10 μM, mucosal side), cyclooxygenase inhibitor indomethacin (10 μM, mucosal and serosal sides), and nerve-conduction blocker tetrodotoxin (300 nM, serosal side) were added to the bathing solution. The activity of ^22^Na was determined in a gamma counter (Cobra 5002, Meriden, CT, USA). The activity of ^36^Cl was counted in a liquid scintillation counter (LSC-3100, Aloka, Tokyo, Japan), and the counts expected from ^22^Na radioactivity were then subtracted from the total counts to yield the count due to ^36^Cl alone, following the method described previously [27].

### Measurement of the alkaline secretion rate

For analysis of HCO_3_^−^ secretion, the isolated and muscle-removed tissues were mounted vertically between Ussing chambers as described above but without short-circuit conditions. The alkaline secretion rate (J(OH)) was determined by continuously titrating mucosal bathing solution to pH 7.2 with 3 mM H_2_SO_4_ under the automatic control of an ABT-101 pH–stat system (TOA Electronics, Tokyo, Japan). The Cl^−^- and buffer-free solution was used for the mucosal solution, which contained (in mM); 140 Na-gluconate, 5.4 K-gluconate, 8 Ca-(gluconate)_2_, 1.2 Mg-(gluconate)_2_ and 10 glucose (gassed with 100% O_2_). The serosal HCO_3_^−^-containing solution is the same as in the Cl^−^ and Na^+^ flux measurements. The J(OH) in the absence of mucosal Cl^−^ was determined first, and then, the mucosal solution was replaced by one containing 75 mM Cl^−^. The mucosal Cl^−^-containing solution had the same composition as the Cl^−^-free solution, except that 75 mM Na-gluconate was replaced by 75 mM NaCl (thus, containing 65 mM Na-gluconate). Finally, the propionate-induced alkaline secretion rate was measured. The mucosal propionate-containing solution had the same composition as the Cl^−^-containing solution except that 65 mM Na-gluconate was replaced by 65 mM Na-propionate. When the pH value of the mucosal solution had decreased (net acid secretion), the acid secretion rate was calculated from the decrease in pH and the titration curve of the mucosal solution, the result being expressed as –J(OH).

### Fecal water content, ion concentrations, and pH

Stools immediately after they were defecated or luminal stool specimens removed from the ileum, the cecum, the proximal colon (0–30% length of entire colon), and the middle-distal colon immediately after sacrifice were placed in preweighed centrifuge tubes. The wet weight of each tube was measured using an electronic analytical balance. For measurement of ion concentrations and pH, the wet samples were suspended in water and centrifuged. The resultant supernatants were used for measurements. The Na^+^ and K^+^ concentrations were measured using an ion electrode (Cardy meter, Horiba, Kyoto, Japan). The Cl^−^ concentrations were measured using a salt analyzer (SAT-210; TOA Electronics, Tokyo, Japan). The sample pH was determined using an Ion Sensitive Field Effect Transistor pH meter (KS723, Shindengen Electric, Tokyo, Japan). After drying the pellet in an oven at 80 °C for 24 h, the dry weight was obtained, and the water content of each sample was calculated from the wet and dry weights.

### Chemicals

We purchased ^36^Cl from Amersham Bioscience (Piscataway, NJ, USA) and ^22^Na from PerkinElmer (Boston, MA, USA). Tetrodotoxin was purchased from Calbiochem (San Diego, CA, USA), and indomethacin and benzamil were purchased from Sigma (St. Louis, MO, USA). Indomethacin, benzamil and tetrodotoxin were supplied from aqueous stock solutions.

### Statistical analysis

Experimental values are given as the mean ± SE. The data were analyzed by ordinary one-way ANOVA with Tukey’s post hoc analysis for multiple comparisons. Comparisons between two groups were made with either unpaired or paired Student’s *t* test, as appropriate. *P* values < 0.05 were considered to be statistically significant. Statistical analysis was carried out with GraphPad Prism 6 (San Diego, CA, USA).

## Results

### Defecated stool compositions: genotype difference

**Fig. 1:**
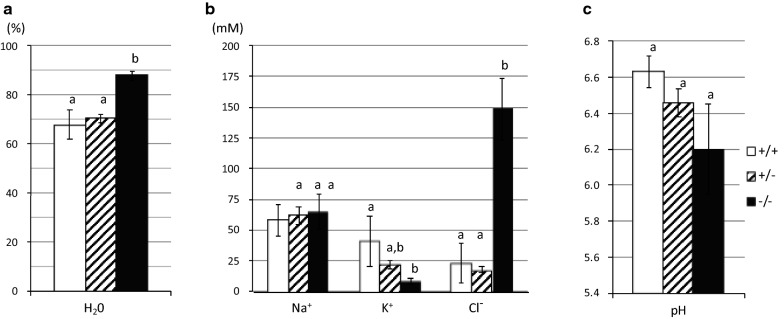
The compositions of defecated stool in wild-type [slc26a3(+/+), open bar], heterozygous [slc26a3(+/−), hatched bar], or DRA-KO [slc26a3(−/−), closed bar] animals; **a** H_2_O concentration, **b** Na^+^, K^+^, and Cl^−^ concentrations, and **c** pH. Statistical comparisons were performed among three different slc26a3 genotypes by ordinary ANOVA with Tukey’s multiple comparisons test. The bars labeled with different letters are significantly different from each other. *n* = 3 for the slc26a3(+/+), *n* = 10 for the slc26a3(+/−) and *n* = 3 for the slc26a3(−/−)

We gathered the stools immediately after they were defecated from wild-type [(slc26a3(+/+)], heterozygous [slc26a3(+/−)], or DRA-KO [slc26a3(−/−)] mice, and measured their H_2_O, Na^+^, K^+^, and Cl^−^ concentrations and pH (Fig. 1). The stool of the DRA-KO mice had markedly elevated H_2_O and Cl^−^ concentrations compared with the stool of the slc26a3(+/+) or slc26a3(+/−) mice. The results also revealed that the Cl^−^ and H_2_O concentrations are similar between the slc26a3(+/+) and slc26a3(+/−) mice. Therefore, we assumed that the functional level of DRA is not markedly different in these two different groups. In most of the following experiments, we grouped the slc26a3(+/+) and slc26a3(+/−) mice together as the control, and the data obtained from the control mice were compared with those from the scl26a3(−/−) (DRA-KO) mice. [Actually, the results obtained from the slc26a3(+/−) mice were not markedly different from those obtained from the slc26a3(+/+) mice as shown in “additional information” of the figure and table legends.]

### Cl^−^ and Na^+^ transport and electrical parameters

**Fig. 2:**
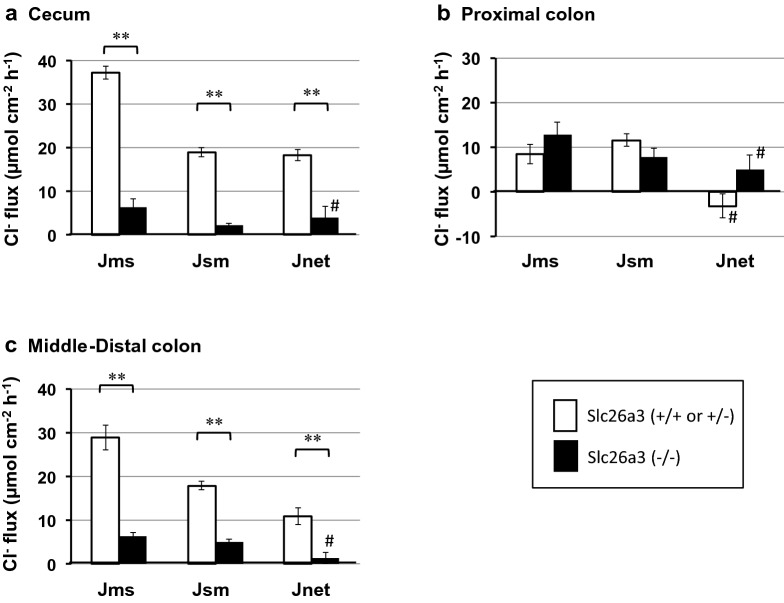
Unidirectional fluxes of ^36^Cl from mucosal to serosal solution (Jms) and serosal to mucosal solution (Jsm) were determined using two adjacent tissues under short-circuit conditions, Jnet being Jms minus Jsm values. The fluxes in the cecum (**a**), proximal colon (0–30% length of entire colon) (**b**), and middle-distal colon (**c**) were compared between the slc26a3(+/+ or +/−) (unfilled square) and slc26a3(−/−) (filled square) mice. These experiments were conducted in the presence of benzamil (10 μM, mucosal side) to suppress electrogenic Na^+^ absorption, in addition to TTX and indomethacin (see the Materials and methods). The results are presented as µEq cm^−2^ h^−1^. Data are the mean ± SE. Statistical comparisons were performed by paired or unpaired *t* test. **p < 0.01 between slc26a3(+/+ or +/−) and slc26a3(−/−). ^#^Not significantly different from zero. *n* = 4 for the slc26a3(+/+ or +/−) mice and *n* = 5–6 for the slc26a3(−/−) mice. Additional information; the numbers of the slc26(+/−) [vs. slc26a3(+ /+)] in the control group are 1 (vs. 3) in the cecum, 0 (vs. 5) in the proximal colon, and 1 (vs. 3) in the middle-distal colon. Jms, Jsm, and Jnet from the slc26(+/−) were 36.7, 17.9, and 18.8 in the cecum, and 25.6, 16.2, and 9.4 in the middle-distal colon [in µEq cm^−2^ h^−1^)]

We used Ussing chambers under short-circuit conditions and measured unidirectional ^36^Cl^−^ and ^22^Na^+^ fluxes in three segments of the large intestine. As shown in Fig. 2, Jms(Cl) was larger than Jsm(Cl); therefore, Jnet(Cl) was positive in the cecum (Fig. 2a) and mid-distal colon (Fig. 2c) of the control mice, indicating the existence of Cl^−^ absorption in these segments. In the DRA-KO mice, Jnet(Cl) was abolished in both the cecum and middle-distal colon, indicating that DRA is expressed and plays a major role in Cl absorption in these segments, in agreement with previous studies [15, 16, 24, 28, 29]. Notably, DRA-KO caused not only a decrease in Jms(Cl) but also a significant decrease in Jsm(Cl) (see the “Discussion” section). In the proximal colon, in contrast to the cecum and middle-distal colon, Jnet(Cl) was low, and Jms(Cl) and Jsm(Cl) were both not changed after DRA-KO (Fig. 2b), suggesting that DRA is barely expressed in this segment, consistent with previous reports [24, 26].

**Fig. 3:**
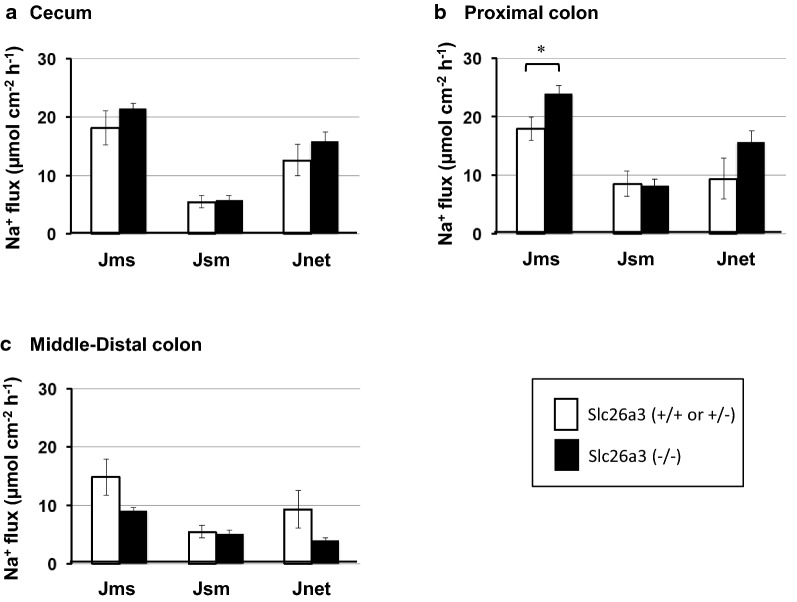
Unidirectional fluxes of ^22^Na from mucosal to serosal solution (Jms) and serosal to mucosal solution (Jsm) were determined using two adjacent tissues under short-circuit conditions, Jnet being Jms minus Jsm values. The fluxes in the cecum (**a**), proximal colon (0–30% length of the entire colon) (**b**), and middle-distal colon (**c**) were compared between the slc26a3(+/+ or +/−) (unfilled square) and slc26a3(−/−) (filled square) mice. These experiments were conducted in the presence of benzamil (10 μM, mucosal side) to suppress electrogenic Na^+^ absorption. The results are presented as µEq cm^−2^ h^−1^. Data are the mean ± SE. Statistical comparisons were performed by unpaired *t* test. *0.01 < *p* < 0.05 between slc26a3(+/+ or +/−) and slc26a3(−/−). *n* = 4 for the slc26a3(+/+ or +/−) mice and *n* = 4–5 for the slc26a3(−/−) mice. Additional information; the numbers of the slc26(+/−) [vs. slc26a3(+/+)] in the control group are 1 (vs. 3) in the cecum, (0 vs. 5) in the proximal colon and 0 (vs. 4) in the middle-distal colon. Jms, Jsm, and Jnet from the slc26(+/−) were 14.8, 2.5 and 12.3 in the cecum (in µEq cm^−2^ h^−1^)

The Jms(Na) values were larger than the Jsm(Na) values, and the Jnet(Na) values were positive in all three segments, indicating that a strong Na absorptive function is present throughout the entire length (Fig. 3). In the DRA-KO mice, Na absorption was preserved in all three segments, particularly in the proximal colon, where Na absorption increased mainly due to the increase in Jms(Na) (Fig. 3b).

The Isc and Gt obtained in the experiments described in Figs. 2 and 3 are summarized in Table 1. It was found that the baseline Isc in the DRA-KO mice was larger than that in the control mice in the cecum and middle-distal colon and possibly also in the proximal colon, a similar result having been reported previously [15, 29]. However, Gt was not significantly different in all three segments between the DRA-KO mice and the slc26a3(+/+ or +/−) control mice.

**Table 1:** Comparison of slc26a3(+/+ or +/−) and slc26a3(−/−) for electrical parameters: short-circuit current (Isc) and transmucosal conductance (Gt)

	slc26a3(+/+ or +/−)	slc26a3(−/−)	*p* values
Cecum			
Isc	0.28 ± 0.13	1.45 ± 0.29	0.007*
Gt	17.2 ± 2.2	13.1 ± 0.9	0.096
Proximal colon			
Isc	1.21 ± 0.25	1.92 ± 0.20	0.059
Gt	19.7 ± 2.6	17.9 ± 2.4	0.633
Middle-distal colon			
Isc	0.64 ± 0.22	1.37 ± 0.10	0.010*
Gt	13.7 ± 1.5	12.3 ± 0.7	0.396

These values were determined in the experiments shown in Figs. 2 and 3. The Isc, with a positive sign representing net positive charge flowing from mucosa to serosa, is presented as µEq cm^−2^ h^−1^, and Gt as mS cm^−2^. Statistical comparisons were performed by unpaired *t* test

*0.01 < *p* < 0.05 between slc26a3(+/+ or +/−) and slc26a3(−/−). *n* = 5 for slc26a3(+/+ or +/−) and 5–6 for slc26a3(−/−). [Additional information; numbers slc26a3(+/−)] vs. slc26(+/+)] in the control group are 1 (vs. 4) in the cecum, 0 (vs. 5) in the proximal colon and 1 (vs. 4) in the middle-distal colon. Isc and Gt from slc26a3(+/−) were 0.16 and 12.0 in the cecum, and 0.46 and 10.5 in the middle-distal colon (in µEq cm^−2^ h^−1^, and mS cm^−2^).]

### Alkaline secretion rate

**Fig. 4:**
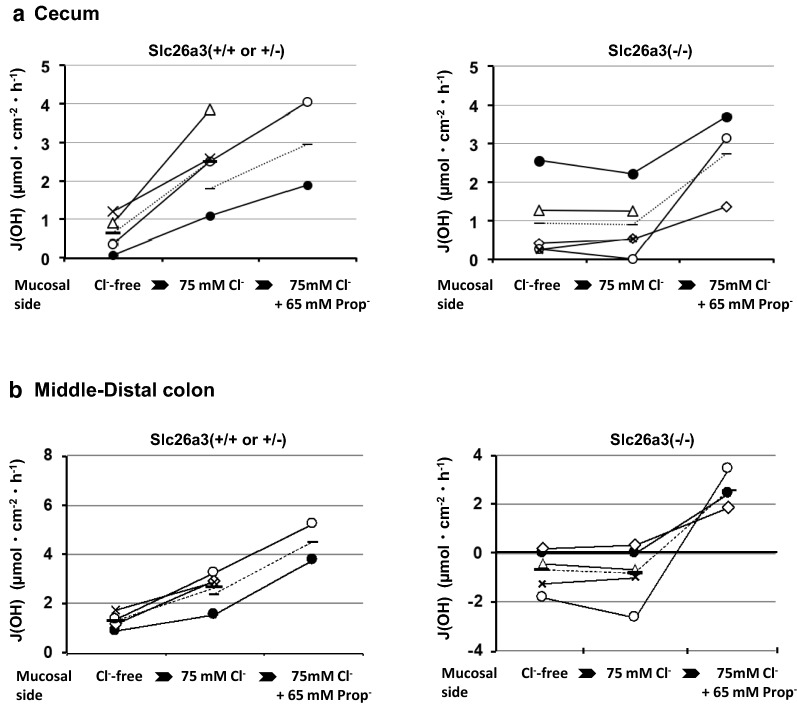
The alkaline secretion rates (J(OH)) induced by Cl^−^ and by propionate in the mucosal solution. J(OH) values were measured by titrating mucosal solution with a pH stat device in the isolated cecum (**a**) or middle-distal colon (**b**) mounted in Ussing chambers. The serosal side was always bathed with HCO_3_^−^-containing buffered solutions, the same solution as used for flux measurements. Left-hand figures; slc26a3(+/+ or +/−) and right-hand figures; slc26a3(−/−). J(OH) was first measured under Cl^−^-free and then 70 mM Cl^−^-containing mucosal solution to determine the Cl^−^-induced HCO_3_^−^ secretion rate. In some tissues, J(OH) was additionally measured under 70 mM Cl^−^ plus 65 mM propionate-containing mucosal solution to determine the propionate-dependent HCO_3_^−^ secretion rate. The same symbols between the cecum and mid-distal colon indicate that tissues were obtained from the same animals. Horizontal bars connected with a dotted line are the mean values. In the middle-distal colon, but not in the cecum, the J(OH) under Cl^−^-free conditions was significantly different between the two groups (unpaired *t* test, *p* < 0.01). The results are presented as µEq cm^−2^ h^−1^. [Additional information; in the left figures the filled circles are slc26a3(+/−), while other symbols are slc26a3(+/+).]

Three HCO_3_^−^ secretion pathways have been suggested to be present in the large intestine: (1) luminal-Cl-dependent secretion, (2) luminal short-chain fatty acid-dependent secretion, and (3) cAMP-stimulated secretion [17, 30–32]. We examined the roles of DRA in the former two HCO_3_^−^ secretion pathways by measuring the alkaline secretion rates (J(OH)) in vitro (Fig. 4). The J(OH) was stimulated by mucosal Cl^−^ in both the cecum and the middle-distal colon. The Cl-dependent increase in J(OH) was largely suppressed in the DRA-KO mice, indicating that DRA is responsible for Cl-dependent HCO_3_^−^ secretion.

The present results, in addition, revealed that J(OH) under Cl-^−^free conditions was significantly reduced in the middle-distal colon, but not the cecum, of the DRA-KO mice (Fig. 4b). This finding could be explained by the increased H^+^ secretion due to the reported upregulation of the H^+^, K^+^-ATPase localizing in the middle-distal colon in the DRA-KO mice [9].

In contrast to the abolishment of Cl-dependent J(OH), the J(OH) induced by mucosal propionate (a short-chain fatty acid) remained after DRA-KO, excluding the direct role of DRA in short-chain fatty acid-dependent HCO_3_^−^ secretion.

### Luminal stool compositions: segmental differences

**Fig. 5:**
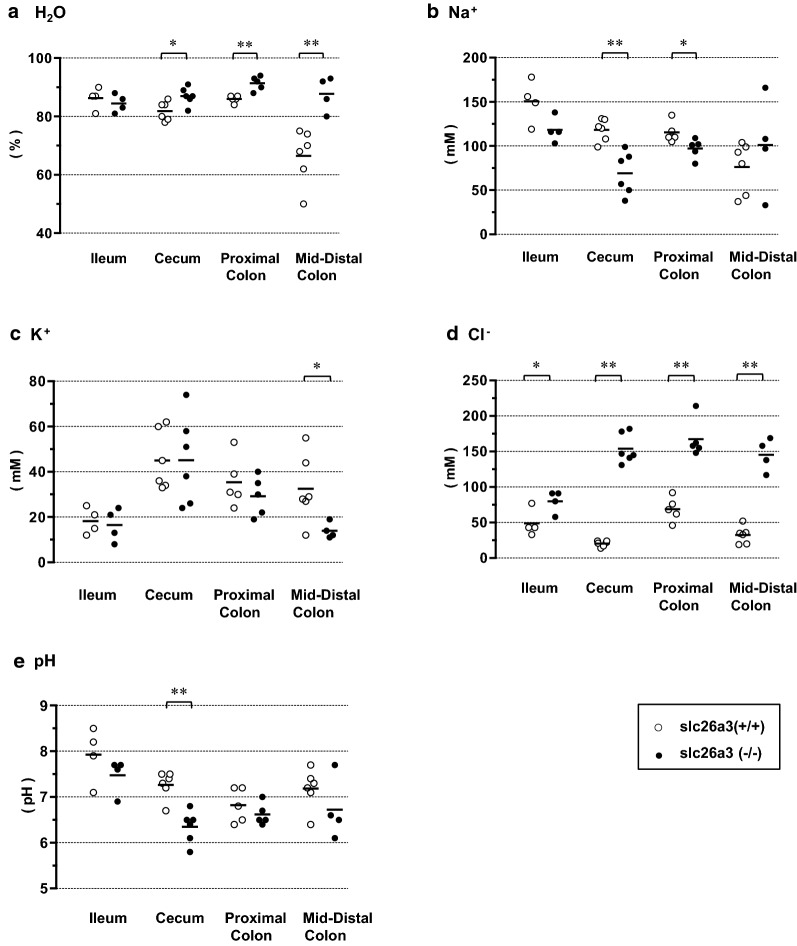
The luminal stool compositions; **a** H_2_O content, **b** Na^+^, **c** K^+^, **d** Cl^−^concentrations, and **e** pH. Open circle; slc26a3(+/+), wild-type mice. Filled circles; slc26a3(−/−), DRA-KO mice. Immediately after the intestine was isolated, a specimen of luminal content was collected from each segment: ileum (4–7 cm proximal to the ileocecal junction), cecum, proximal colon, and middle-distal colon. Statistical comparisons were performed by unpaired *t* test. *0.01 < *p* < 0.05 and **p < 0.01

We obtained luminal stool specimens separately from the ileum, cecum, proximal colon, and middle-distal colon in both the wild-type [slc26a3(+/+) and DRA-KO (slc26a3(−/−)] mice (Fig. 5). The H_2_O content of the luminal stool obtained from the slc26a3(+/+) mice showed a marked decrease (dehydration) in the segment distal to the proximal colon (Fig. 5a open circle), similar to previous observations in the rat large intestine [24]. In the DRA-KO mice, the H_2_O contents of the luminal stools were significantly higher than those in the wile-type mice, and the notable stool dehydration in the middle-distal colon was abolished (Fig. 5a filled circle). Therefore, DRA plays an important role in water absorption in the cecum and particularly in the middle-distal colon, as reported previously [17].

The stool Na^+^ concentration of the wild-type mice gradually decreased from the ileum to the middle-distal colon (Fig. 5b open circle). In the DRA-KO mice, the Na^+^ concentrations in the cecum and proximal colon were significantly lower than those in the wild-type mice (Fig. 5b filled circle).

The stool K^+^ concentrations in the large intestine were generally higher than those in the serum (Fig. 5c open circle). In the DRA-KO mice, the K^+^ concentrations in the middle-distal colon were slightly but significantly lower than those in the wild-type mice (Fig. 5c filled circle). This finding could be explained by the increased K^+^ absorption due to upregulation of H^+^, K^+^-ATPase in the middle-distal colon of the DRA-KO mice, as reported previously [9].

The Cl^−^ concentration profile along the large intestine of the wild-type mice indicated that it was higher in the proximal colon than in the cecum or in the middle-distal colon (Fig. 5d open circle). In the DRA-KO mice, consistent with a previous report [9], the stool Cl^−^ concentrations were markedly elevated in all segments of the large intestine (Fig. 5d filled circle).

The pH of the luminal stool in the wild-type mice was higher in the cecum and the middle-distal colon than in the proximal colon (Fig. 5e, open circle), consistent with previous reports [14, 23, 24]. In the DRA-KO mice, the stool pH was decreased in the cecum but not in the proximal and middle-distal colons (Fig. 5e filled circle).

The ileal stool compositions were consistent with a finding that DRA is also expressed to some extent in the small intestine [10]. The marked difference in stool compositions in the cecum compared with those in the ileum indicates that the changes in stool composition in the cecum after DRA-KO are not merely due to the inflow of the altered stool from the ileum but mostly due to the changes in mucosal functions in this segment.

## Discussion

**Fig. 6:**
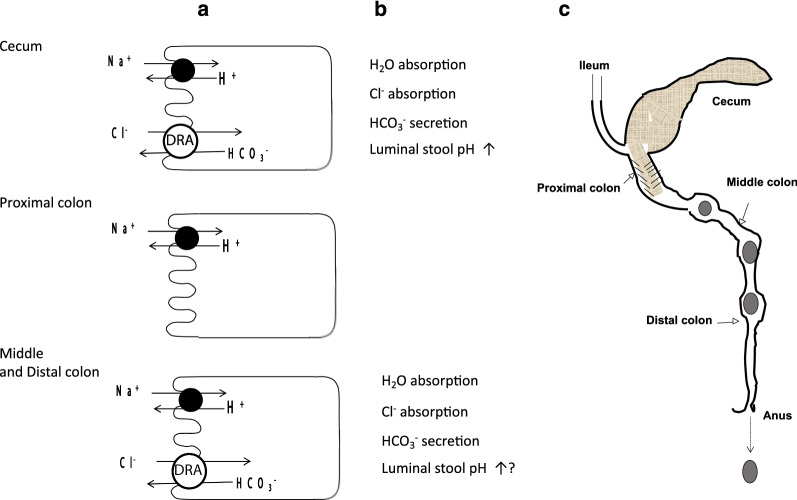
The mucosal transport activities (**a**), the roles of DRA (**b**), and a picture of the mouse distal intestine (**c**)

DRA (SLC26A3) is highly expressed in the large intestine [9, 10], but its expression is heterologous along the cecocolonic axis in the rodent large intestine; DRA is expressed in the cecum and middle-distal colon but not in the early proximal colon [24–26]. The present study, therefore, separately examined the involvement of DRA in Cl^−^ and Na^+^ absorption, HCO_3_^−^ secretion, and luminal electrolyte and water compositions in these three segments of the mouse large intestine. The main findings are summarized in Fig. 6.

The present results (Fig. 2) showed that robust Cl^−^ absorptions which were largely abolished after DRA deficiency were present in both the cecum and the middle-distal colon, but not in the proximal one-third of the colon. The present finding together with these previous findings [15, 16, 24, 26, 28, 29, 33, 34] strongly indicate that DRA is expressed in the cecum and middle-distal colon, and plays a major role in Cl^−^ absorption in these segments of the mouse large intestine. This finding may also be true for the rat large intestine, since Cl^−^ absorptive activity in the rat large intestine has been observed in the cecum [35] and distal colon [22, 36, 37] but not in the early proximal colon [22], consistent with the DRA expression profile of the rat large intestine [24]. DRA-dependent Cl^−^ absorption has a major role in lowering Cl^−^ concentration in the luminal stool. The ablation of DRA increased the stool Cl^−^ concentration in the large intestine of the DRA-KO mice (this study, 9) and in the feces of CCD patients [2–4]. The stool Cl^−^ concentration in the proximal colon, a segment where DRA is barely expressed, was significantly higher compared than that in the cecum and the middle-distal colon in mice (this study) and rats [24].

One intriguing finding obtained by ^36^Cl^−^ flux experiments is that after DRA-KO, not only Jms(Cl) but also Jsm(Cl) was significantly decreased in both the cecum and mid-distal colon, agreeing with several previous reports [15, 29]. Similarly, a decrease in ^36^Cl flux into the perfused colon has been reported in CCD patients [6]. The decrease in a component of Jsm(Cl) after DRA ablation is probably mediated through a cellular pathway, since it was reported to be inhibited by DIDS [15]. In addition, Gt or Jsm(Na) did not change after DRA-KO (this study, [15, 29]), excluding changes in a Jsm(Cl) component through paracellular pathways. The apical exit process of this Jsm(Cl) component could involve DRA through Cl^−^/Cl^−^ exchange activity [12] or by CFTR Cl^−^ channel supposedly associated with DRA [15].

This study demonstrated Na^+^ absorption in the cecum, proximal colon, and middle-distal colon in mice. These processes are likely mediated mainly by the electroneutral sodium/proton exchanger 3 (NHE3) in the apical membrane; the experiments were performed while inhibiting the electrogenic ENaC-mediated Na^+^ absorption. Electroneutral Na^+^ absorption activity has been demonstrated previously in the cecum [33] and proximal colon [34, 38] of mice and in the cecum [35] and in the early proximal colon and distal colon [22, 37] of rats. Significant NHE3 mRNA has been previously observed in the cecum and colon of mice [39, 40]. However, an immunodetection study failed to detect apical NHE3 protein in the cecum and distal one-third of the colon of rats and mice [24]; this discrepancy remains to be explained. NHE3 expression and activity have been reported to be enhanced in DRA-deficient mice [9, 17]). The present study showed that after DRA-KO, Jnet(Na) indeed increased mainly due to the increase in Jms(Na) in the proximal colon. However, Jnet(Na) did not change in the cecum and seemed to decrease in the mid-distal colon (Fig. 3). The absence of increases in Jnet(Na) in these segments is possibly explained by NHE3-mediated Na^+^ absorption, which is more or less coupled to DRA activity [41]; therefore, the increase in NHE3 expression was compromised by DRA deletion. The interaction between NHE3 and DRA remains to be elucidated.

It has been reported that luminal-Cl^−^ dependent HCO_3_^−^ secretion is highly active in the cecum and middle-distal colon but minimal in the proximal one-third of the colon in mice, in parallel with DRA expression in these segments [14, 23, 24]. This and previous studies have shown that DRA is responsible for Cl^−^-dependent HCO_3_^−^ secretion in both the cecum (this study) and the middle-distal colon (this study, 14, 17). HCO_3_^−^ secretion would cause an increase in stool pH. The decreased stool pH after DRA-KO in the cecum can be explained by elimination of DRA-induced HCO_3_^−^ secretion. The lower stool pH values in the proximal one-third of the colon of wild-type mice (this study) and rats [24] are similarly explained by the absence of DAR-mediated HCO_3_^−^ secretion in this segment. DRA-KO failed, however, to lower the pH of the stool in the middle-distal colon (Fig. 5) and defecated stools (Fig. 1). This finding is even more strange, because H^+^ secretion mediated by H^+^, K^+^-ATPase was upregulated after DRA-KO (see above and 9). Thus, luminal pH regulation in the middle-distal colon is a sophisticated process involving NHE, DRA, H^+^, K^+^-ATPase, and possibly additional acid/base regulators.

## Conclusions

This study demonstrated that DRA (slc26a3) mediates Cl^−^ absorption and HCO_3_^−^ secretion in the mouse cecum and middle-distal colon, consistent with the previous reports [15, 16, 24, 26, 28, 29, 33, 34]. In addition, we, for the first time, demonstrated that (1) Cl-dependent HCO_3_^−^ secretion is absent in the DRA-deficient cecum, (2) DRA is not responsible for short-chain fatty acid-dependent HCO_3_^−^ secretion, and (3) Na absorption is preserved in the cecum and middle-distal colon and probably enhanced in the proximal colon of the DRA-deficient mice. In addition, 4) we determined stool compositions in each segment of the large intestine in both the wild-type and DRA-deficient mice to elucidate the influence of DRA on stool composition. A limitation of the present study is that we used both slc26a3(+/+) and slc26a3(+/−) mice as controls and compared them with slc26a3(−/−) mice in the Ussing chamber experiments. A possible difference between the slc26a3(+/+) or slc26a3(+/−) mice in transport activities could not be ignored. Finally, the physiological and pathological significance of the absence of DRA in the proximal one-third of the colon remains to be investigated, in view of the recent evidence for mucosal protective roles of DRA-dependent HCO_3_^−^ secretion [17, 42].

## Data Availability

The datasets used and/or analyzed during the current study are available from the corresponding author on reasonable request.
